# Socioeconomic inequalities in self-rated health in Japan, 32 European countries and the United States: an international comparative study

**DOI:** 10.1177/14034948221092285

**Published:** 2022-05-10

**Authors:** Hirokazu Tanaka, Wilma J. Nusselder, Yasuki Kobayashi, Johan P. Mackenbach

**Affiliations:** 1 Department of Public Health, Erasmus University Medical Center, Rotterdam, The Netherlands; 2 Department of Public Health and Occupational Medicine, Mie University, Mie, Japan; 3 Department of Public Health, Graduate School of Medicine, the University of Tokyo, Tokyo, Japan

**Keywords:** Self-assessed health, perceived health, health surveys, socioeconomic factors

## Abstract

**Aims:**

Japan is known as a country with low self-rated health despite high life expectancy. We compared socioeconomic inequalities in self-rated health in Japan with those in 32 European countries and the US using nationally representative samples.

**Methods:**

We analysed individual data from the Comprehensive Survey of Living Conditions (Japan), the European Union Statistics on Income and Living Conditions, and the Behavioral Risk Factor Surveillance System (US) in 2016. We used ordered logistic regression models with four ordinal categories of self-rated health as an outcome, and educational level or occupational class as independent variables, controlling for age.

**Results:**

In Japan, about half the population perceived their health as ‘fair’, which was much higher than in Europe (≈20–40%). The odds ratios of lower self-rated health among less educated men compared with more educated were 1.72 (95% confidence interval (CI) 1.61–1.85) in Japan, and ranged from 1.67 to 4.74 in Europe (pooled; 2.10 (95% CI 2.01–2.20)), and 6.65 (95% CI 6.22–7.12) in the US. The odds ratios of lower self-rated health among less educated women were 1.79 (95% CI 1.65–1.95) in Japan, and ranged from 1.89 to 5.30 in Europe (pooled; 2.43 (95% CI 2.33–2.54)), and 8.82 (95% CI 8.29–9.38) in the US. Socioeconomic inequalities were large when self-rated health was low for European countries, but Japan and the US did not follow the pattern.

**Conclusions:**

**Japan has similar socioeconomic gradient patterns to European countries for self-rated health, and our findings revealed smaller socioeconomic inequalities in self-rated health in Japan compared with those in western countries.**

## Introduction

Assessment of individual own health, that is self-rated health (SRH; also known as self-assessed health or self-perceived health), reflects the quality of life in terms of both mental and physical health. SRH has been shown to be a reliable indicator of general health [1, 2] and a predictor of future mortality [3, 4]. Ratings of SRH by survey participants are often based on responses to a single question (‘How is your health, in general?’), and are widely used in population health monitoring. Cross-national comparisons using SRH as an outcome offer excellent opportunities for assessing health and socioeconomic inequalities in different regions.

Reducing socioeconomic inequalities in health is a key public health priority globally; this includes achieving equalities both within a country and across countries [5]. In Europe, for example, numerous studies have compared health inequalities across neighbouring countries; there are clear geographical variations in health inequalities [6–10]. The results of those researches showed that the magnitudes of inequalities in mortality were relatively small in southern European countries (i.e. Italy and Spain) and very large in most countries in the eastern European (e.g. Hungary and Czech Republic) and Baltic regions [6–8]. These findings have contributed to interesting discussions about, for example, the Nordic paradox: Scandinavian countries appear to have comparatively large health inequalities, although these countries have the most generous welfare arrangements in the world, reflecting long histories of egalitarian policies [11]. However, intercontinental comparative studies on health inequalities, especially looking at the differences between countries with a ‘Confucian’ welfare regime (e.g. Japan [12]) and western countries remain scarce. By increasing the range of variation in determinants of health inequalities, such intercontinental comparisons can provide important insights into the determinants of health inequalities, and thereby help us to determine what further actions to take to reduce health inequalities.

Japan has achieved very high life expectancy [13]; however, SRH was reported to be less favourable than any other high-income country except South Korea in the 2010s [14]. According to the statistics provided by the Organisation for Economic Co-operation and Development (OECD), although 8.7% of adults consider themselves to be in poor health on average across 35 OECD countries in 2017, the percentage was higher (14.1%) with small income-based inequalities in Japan [14]. The unique patterns in terms of high life expectancy but low SRH in Japan (‘high life expectancy but low SRH’ paradox) may be comparable to those observed in its neighbour, South Korea [15, 16]. Therefore, more definitive evidence regarding how the magnitude of inequalities in SRH in Japan compares with that in other high-income countries is needed.

Within this context, this study systematically compared socioeconomic inequalities in SRH between Japan, 32 European countries, and the US using the national representative data. We aimed to investigate whether the pattern and the magnitude of socioeconomic inequalities in Japan were comparable with those in European countries and the US.

## Methods

### Data

We obtained nationally representative individual data from the Comprehensive Survey of Living Conditions (CSLC) in Japan [17], the European Union (EU) Statistics on Income and Living Conditions (EU-SILC) [18], and the Behavioral Risk Factor Surveillance System (BRFSS) in the US [19] as of 2016, respectively. The CSLC has been conducted by the Japanese Ministry of Health, Labor, and Welfare (MHLW) since 1986 to survey income, living conditions, health status, and medical-seeking and caregiving behaviours [17]. The EU-SILC collects comparable cross-sectional and longitudinal data on income, poverty, social exclusion, and living conditions every year. We included all 28 EU member countries (as of 2016) plus Norway, Switzerland, Serbia, and Iceland [18]. The BRFSS are health-related surveys that collect data about US residents regarding their health-related risk behaviours, chronic health conditions, and use of preventive services.

Microdata from the CSLC and EU-SILC were extracted and used with permission from the MHLW and Eurostat, respectively. Microdata from the BRFSS were downloaded because they were open data [19]. The overview of survey data is shown in Table 1. Stata version 15.0 (Stata Corp, College Station, TX, USA) was used for the data management and statistical analysis.

### Definition of SRH

The response scale was symmetrical in Japan (CSLC) and European countries (EU-SILC), whereas the response scale used in the US (BRFSS) was asymmetrical (skewed on the positive side) [17–19]. The CSLC and EU-SILC asked about respondents’ SRH status with the single question in each native language: ‘How is your health in general? Is it very good, good, fair, bad, or very bad’ [17, 18]? The BRFSS asked a similar question: ‘Would you say that in general your health is: excellent, very good, good, fair, poor’ [19]. The differences between these two measures of SRH were discussed in a previous study [20]. We collapsed the five categories of SRH from the question into four ordinal categories of SRH: 0 = very good (excellent and very good for the US); 1 = good; 2 = fair; and 3 = bad/very bad (poor only for the US) as an outcome variable. This is because the prevalence of ‘very bad’ is generally very low in a symmetrical scale [20].

### Socioeconomic status

Socioeconomic status was measured by both educational level and occupational class. Educational level was categorised on the basis of the international standard classification of education (ISCED) and divided into three categories: low (ISCED: 1, 2); middle (ISCED: 3, 4); and high (ISCED: 5–8) (Supplemental Table 1–1) [6, 21–23]. Occupational class was divided into five categories: upper non-manual workers; lower non-manual workers; manual workers; farmers; and the self-employed. This classification followed the Erikson–Goldthorpe–Portocarero (EGP) scheme (Supplemental Table 1–2) [24]. Unemployed and unpaid people (such as stay-at-home spouses and volunteers) were excluded from the analysis regarding occupational class inequalities. Occupational class was not analysed for the US and Iceland due to a lack of data.

### Statistical analysis

All analyses were conducted by sex using the weight score provided by each survey and restricted to survey participants aged 30–79 years. For the pooled analysis of European countries (including 26 EU member countries except for Luxemburg and Malta), we applied the adjusted weight to take the population sizes of each country into account [25]. We used ordered logistic regression models, with ordinal scale SRH as an outcome, and age category (5-year age interval) and educational level or occupational class (restricted to 30–64 years), to determine each variable’s probability of lower SRH [26, 27]. Odds ratios (ORs) for the four-level (0 = very good; 1 = good; 2 = fair; and 3 = bad/very bad) ordered regression model show whether lower SRH compared with the reference socioeconomic group; therefore, higher ORs mean larger socioeconomic inequalities whereas OR≈1 means having no virtually absent socioeconomic differences in SRH. After fitting the model, the age-adjusted percentages of SRH were calculated by estimating the predicted SRH, for each study participant, fixing educational level or occupational class at each categorical level and averaging over the sample [28].

The associations were examined using a univariate linear regression analysis to assess: (a) the pattern of inequalities as a function of educational level and occupational class; and (b) the international patterns regarding the relationship between the percentage of less than good SRH (the sum of all answer categories below ‘good’) and the magnitude of inequalities by educational level. We plotted the associations between the magnitude of inequalities as a function of educational level and those by occupational class; the correlations were evaluated using Pearson’s correlation coefficients by sex to confirm the correlation across European countries. Finally, we plotted the associations between the percentage of less than good SRH and the magnitude of inequalities as a function of educational level.

## Results

### Self-rated health

**Figure 1.**
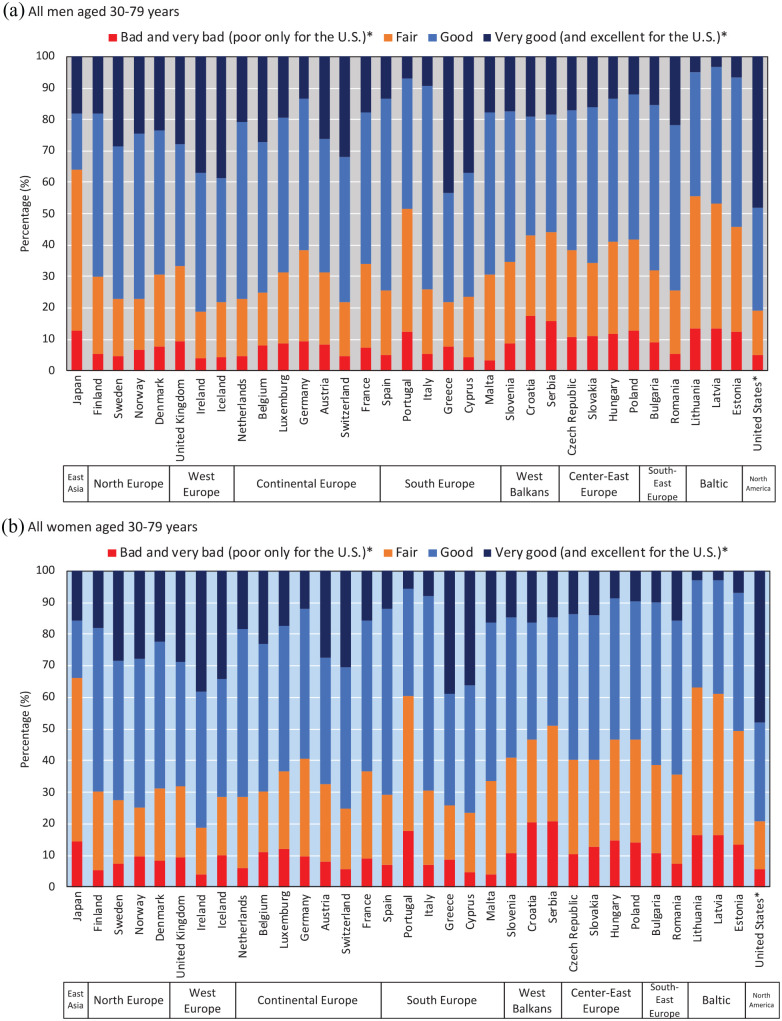
Age-adjusted self-rated health among men and women aged 30–79 years. *The response scale used in the US was asymmetrical (skewed on the positive side), including the response categories: ‘excellent’, ‘very good’, ‘good’, ‘fair’, ‘poor’.

Figure 1 describes age-adjusted percentages of SRH across all countries included in this study. In Japan, age-adjusted percentages of SRH (very good, good, fair, and bad/very bad) were 18.1%, 17.8%, 51.4%, and 12.8% for men aged 30–79 years, whereas the percentages were 17.2%, 50.3%, 24.5%, and 8.0% in Europe (adjusted pooled data). For women, age-adjusted percentages of SRH (very good, good, fair, and bad/very bad) were 15.7%, 18.1%, 51.8%, and 14.3% in Japan, whereas the percentages were 15.5%, 48.3%, 26.8%, and 9.4% in Europe (adjusted pooled data). In the US, age-adjusted percentages of SRH (excellent/very good, good, fair, and poor) were 48.2%, 32.7%, 13.9%, and 5.2% for men and 48.0%, 31.3%, 15.1%, and 5.7% for women, respectively.

**Table I.** Overview of survey data.

Region	Country	Survey name (year)	Number of respondents	Missing^a^	Missing rate (%)	Percentage of low educated	
						Men	Women
East Asia	Japan	CSLC (2016)	376,043	15,933	4.2	12.4	12.4
North Europe	Finland	EU-SILC (2016)	16,226	8215	50.6	17.9	15.2
	Sweden	EU-SILC (2016)	8456	3998	47.3	17.0	18.5
	Norway	EU-SILC (2016)	10,026	4817	48.0	15.2	15.7
	Denmark	EU-SILC (2016)	9252	4180	45.2	17.3	20.2
West Europe	UK	EU-SILC (2016)	13,610	2677	19.7	34.2	34.0
	Ireland	EU-SILC (2016)	7853	0	0.0	37.8	29.1
	Iceland	EU-SILC (2016)	4660	2484	53.3	21.5	28.9
Continental Europe	Netherlands	EU-SILC (2016)	18,683	8331	44.6	19.5	25.8
	Belgium	EU-SILC (2016)	8376	35	0.4	25.8	27.0
	Luxemburg	EU-SILC (2016)	6109	15	0.2	30.1	39.0
	Germany	EU-SILC (2016)	20,297	41	0.2	6.4	13.5
	Austria	EU-SILC (2016)	8437	0	0.0	9.5	21.1
	Switzerland	EU-SILC (2016)	11,437	1452	12.7	6.8	13.6
	France	EU-SILC (2016)	16,358	245	1.5	23.5	28.5
South Europe	Spain	EU-SILC (2016)	23,562	159	0.7	50.0	49.8
	Portugal	EU-SILC (2016)	17,499	8	0.0	71.0	66.1
	Italy	EU-SILC (2016)	32,125	786	2.4	42.1	44.1
	Greece	EU-SILC (2016)	29,054	0	0.0	37.6	43.3
	Cyprus	EU-SILC (2016)	6963	1	0.0	31.9	33.4
	Malta	EU-SILC (2016)	7319	0	0.0	65.7	67.4
West Balkans	Slovenia	EU-SILC (2016)	16,237	9543	58.8	14.7	23.0
	Croatia	EU-SILC (2016)	12,966	103	0.8	21.0	35.2
	Serbia	EU-SILC (2016)	11,477	0	0.0	24.5	35.9
Center-East Europe	Czech Republic	EU-SILC (2016)	12,836	3169	24.7	4.9	12.9
	Slovakia	EU-SILC (2016)	10,556	55	0.5	7.5	12.9
	Hungary	EU-SILC (2016)	12,357	36	0.3	18.2	27.6
	Poland	EU-SILC (2016)	20,798	1750	8.4	13.8	17.3
South-East Europe	Bulgaria	EU-SILC (2016)	12,176	0	0.0	26.4	27.6
	Romania	EU-SILC (2016)	12,422	0	0.0	39.2	49.7
Baltic	Lithuania	EU-SILC (2016)	7411	1984	26.8	9.6	10.7
	Latvia	EU-SILC (2016)	8934	195	2.2	17.0	13.1
	Estonia	EU-SILC (2016)	9256	2377	25.7	22.0	15.4
North America	US^b^	BRFSS (2016)	390,223	5	0.0	8.7	8.2

CSLC: Comprehensive Survey of Living Conditions; EU-SILC: European Union Statistics on Income and Living Conditions; BRFSS: Behavioral Risk Factor Surveillance System.

a Number of respondents (men and women aged 30–79 years) whose self-rated health was not reported.

b The response scale used in the US (BRFSS) was asymmetrical (skewed on the positive side), including the response categories: ‘excellent’, ‘very good’, ‘good’, ‘fair’, and ‘poor’, whereas the response scale was symmetrical in Japan (CSLC) and European countries (EU-SILC), including the response categories: ‘very good’, ‘good’, ‘fair’, ‘poor’, and ‘very poor’.

### Socioeconomic inequalities in SRH

**Figure 2.**
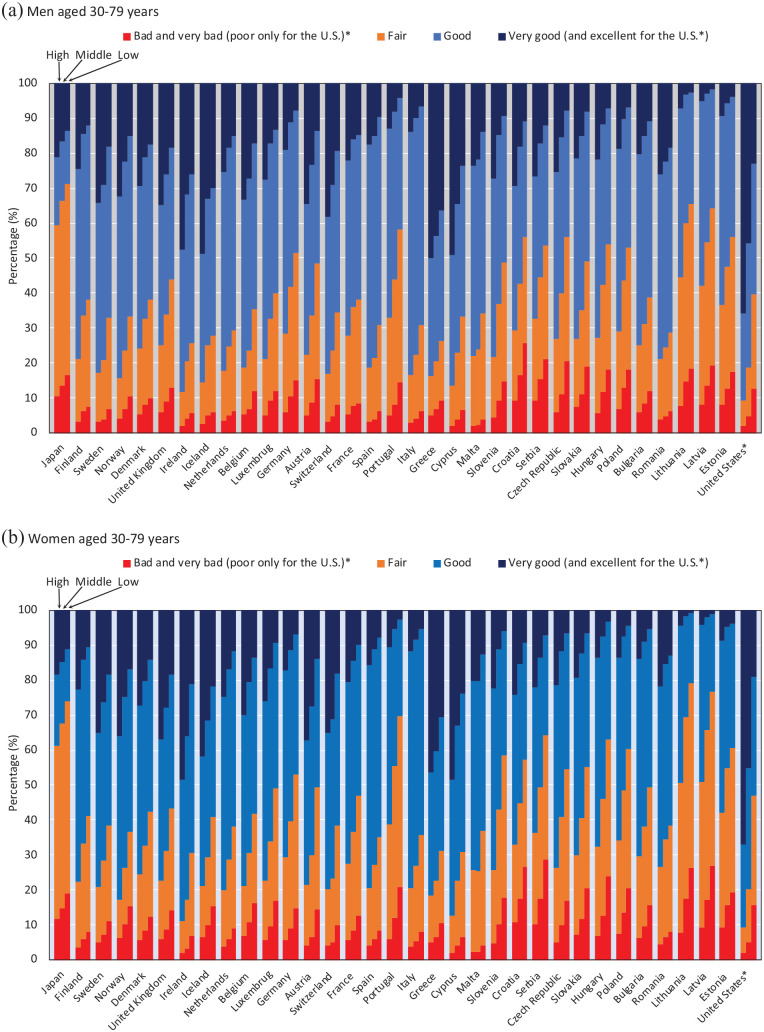
Self-rated health by educational level among men and women aged 30–79 years. *The response scale used in the US was asymmetrical (skewed on the positive side), including the response categories: ‘excellent’, ‘very good’, ‘good’, ‘fair’, ‘poor’.

Figure 2 shows the differences in prevalence of different categories of SRH by educational level across all countries. The prevalence of less than good SRH was 59.3% among highly educated men in Japan, which was even higher than that of European men with low levels of education (adjusted pooled data 36.9%). For women, the prevalence of less than good SRH was 61.3% among highly educated women in Japan, which was even higher than that of women with low levels of education in European countries (adjusted pooled data 42.8%).

**Figure 3.**
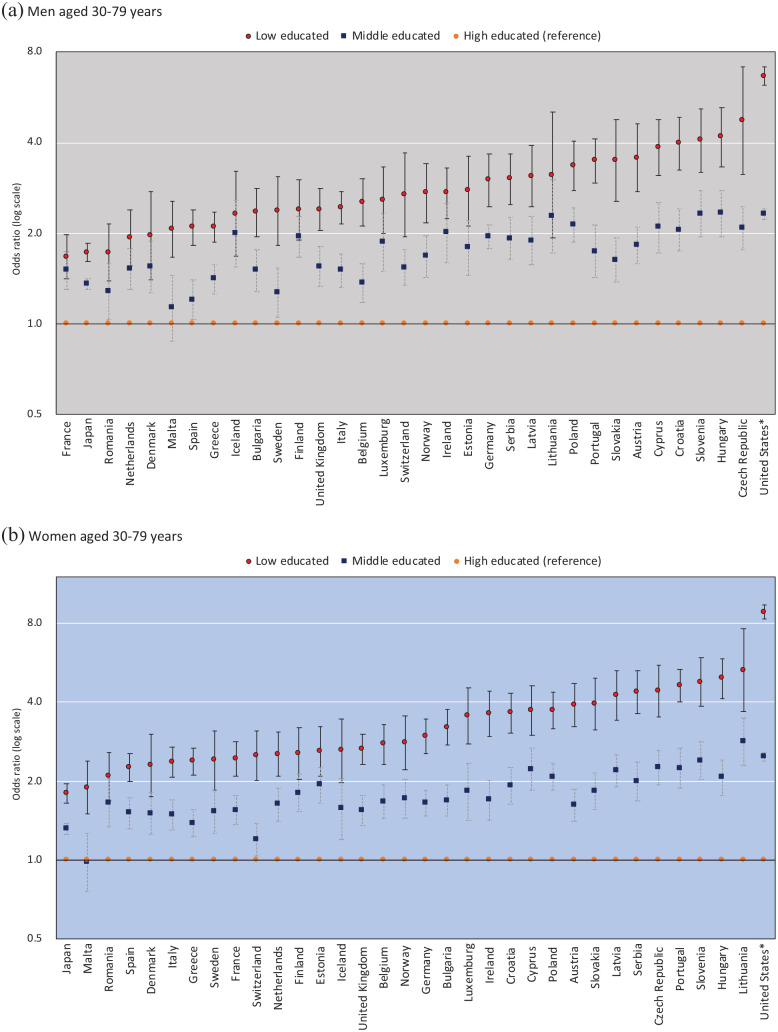
Odds ratios of less good self-rated health by educational level among men and women aged 30–79 years.

Figure 3 shows relative educational inequalities in SRH. Japan had smaller educational inequalities in SRH than those observed in almost all European countries. The ORs of lower SRH among men with low education levels compared with highly educated men were 1.72 (95% confidence interval (CI) 1.61–1.85) in Japan, ranging from 1.67 (95% CI 1.41–1.98) in France to 4.74 (95% CI 3.13–7.17) in the Czech Republic in Europe (pooled data; 2.10 (95% CI 2.01–2.20)), and was 6.65 (95% CI 6.22–7.12) in the US (see also Supplemental Table 1–3). The ORs of lower SRH among women with a low education level compared with highly educated women were 1.79 (95% CI 1.65–1.95) in Japan, ranging from 1.89 (95% CI 1.50–2.38) in Malta to 5.30 (95% CI 3.69–7.61) in Lithuania in Europe (pooled data; 2.43 (95% CI 2.33–2.54)), and was 8.82 (95% CI 8.29–9.38) in the US (see also Supplemental Table 1–4). In Europe, relatively larger inequalities were observed in Portugal, Austria, the western Balkans, eastern Europe (except Romania), and Baltic countries for both sexes while France, Romania, Netherlands, Denmark, and southern Europe (e.g. Italy, Spain, and Greece) had smaller socioeconomic inequalities for both sexes than most other countries. The US was found to have larger socioeconomic inequalities in SRH than those observed in Japan and all European countries, but it should be kept in mind that the response scale was not completely the same.

**Figure 4.**
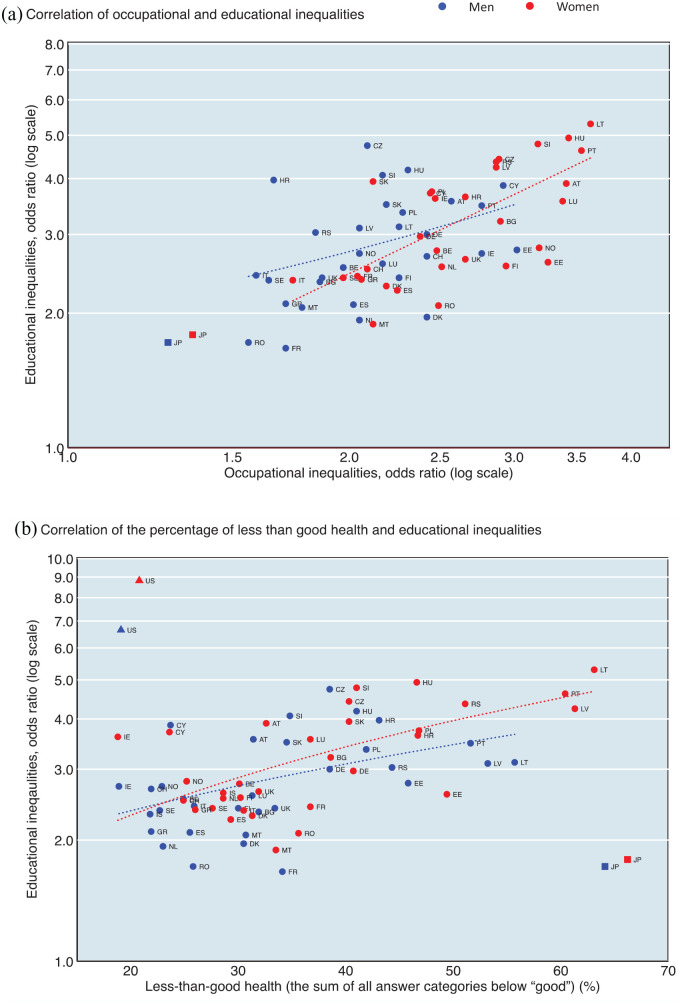
(a) The associations between inequalities by occupational class (manual vs. upper non-manual workers) and inequalities by educational level (low vs. high educational levels) except for the US and Iceland; Pearson’s correlation coefficients: 0.38 (*P*=0.04) for men and 0.67 (*P*<0.01) for women (31 European countries). (b) The associations between the percentage of less than good health and inequalities by educational level (low vs. high educational levels); Pearson’s correlation coefficients: 0.47 (*P*<0.01) for men and 0.66 (*P*<0.01) for women (32 European countries).

Inequalities in SRH by occupational class are shown in Supplemental Tables 1–5 and 1-6. Figure 4(a) shows the correlations between inequalities by occupational class (manual vs. upper non-manual workers) and inequalities by educational level (low vs. high educational levels). The results showed that the overall trends were similar for both educational level and occupational class across countries. Pearson’s correlation coefficients between inequalities by occupational class and educational level for the 31 European countries were 0.38 (*P*=0.04) for men and 0.67 (*P*<0.01) for women, respectively. The magnitude of socioeconomic inequalities was larger among women than men; women were more likely to be in the upper right quadrant of the plot for each country. This figure also confirmed that Japan had smaller socioeconomic inequalities in SRH than all European countries.

Figure 4(b) shows the correlations between the prevalence of less than good SRH and the magnitude of inequalities by educational level. Educational inequalities were more likely to be large when the prevalence of less than good SRH was large for European countries. Pearson’s correlation coefficients for the 32 European countries were 0.47 (*P*<0.01) for men and 0.66 (*P*<0.01) for women, respectively. Interestingly, those of Japan and the US were clearly outliers in comparison to the correlations for European countries. Japan had a high prevalence of less than good SRH with small socioeconomic inequalities whereas the pattern was reversed in the US.

## Discussion

### Summary of main findings

This is the first study that systematically compared socioeconomic inequalities in SRH between Japan, European countries, and the US. Whereas most previous studies used dichotomised SRH as an outcome, we used four categories of SRH, which allowed us to study the full spectrum of SRH. In Japan, about half the population perceived their health as ‘fair’, which was much higher than in Europe (≈20–40%). The prevalence of less than good SRH was even higher among highly educated groups and upper non-manual workers in Japan than among individuals with low education levels and manual workers in Europe, respectively. Smaller socioeconomic inequalities in SRH were observed in Japan than in European countries and the US. Socioeconomic inequalities were large when SRH was low for European countries, but Japan and the US did not follow this pattern.

### Limitations

We aimed to document similarities and differences between three world regions, but we should carefully interpret the results of cross-country comparisons of SRH. The response scale used in the US was asymmetrical (skewed on the positive side), including the response categories ‘excellent’ to ‘poor’, whereas the response scale was symmetrical in Japan and European countries. This difference in measures of SRH may introduce a bias in comparisons across countries, for example, a more positive self-assessment of health in the US [20]. This is likely to be unavoidable in the case of cross-country comparisons using existing national surveys [14]. In particular, the differences between symmetrical scales (used in EU-SILC and Japan) and asymmetrical scales (used in the US and Canada) may lead to problems in international comparability. Because this potentially impedes a detailed intercontinental comparative analysis of SRH, we emphasise that global standardisation of questionnaires in SRH is a key step for better understanding worldwide health inequalities.

SRH responses may also differ across countries because of social and cultural differences. For example, it has been suggested that Japanese people tend to give modest answers irrespective of socioeconomic status in order to avoid extreme responses [29]. This response pattern has been attributed to the ‘collectivism’ that is common in east Asia [30]. The Japanese look positively on the word ‘futsu’ (fair, in Japanese), and therefore often choose the mid-point when they respond to a symmetrical Likert-type scale, even if they have a positive health perception. This may explain why in Japan the prevalence of ‘fair’ SRH is much higher than in European countries and the US [16]. However, it is important to note that our findings also show a higher prevalence of ‘bad’ and ‘very bad’ SRH in Japan than in European countries, suggesting that in Japan the whole distribution is shifted to lower SRH categories (Figures 1 and 2). East Asian (e.g. Japan and South Korea) cultural factors regarding perceived health may affect SRH comparability to western countries. In addition, in Japan, all employers with 50 workers or more must provide general health check-ups once a year to all workers by law; abnormal findings are reported among approximately half of workers, including those that do not require treatment [31]. In other words, health check-ups may paradoxically make workers aware that they are ‘not healthy’. Our findings therefore again illustrate this ‘high life expectancy but low SRH’ paradox in Japan, and this appears to apply to all socioeconomic groups. Further studies are necessary to elucidate whether a higher prevalence of ‘bad’ and ‘very bad’ SRH in Japan is associated with a higher prevalence of objective medical conditions and disease in the Japanese population as compared to European countries.

Our ordered logistic regression analysis aimed to reduce any bias that may be caused, by quantifying the impact of education/occupational class on all steps in the SRH spectrum, not only on the dichotomy between ‘very good’/‘good’ and all categories less than ‘good’. The validity of this method depends on the proportional odds assumption that means similar estimations across cut points [26, 27]. Our analysis suggests that these assumptions were partly violated for some countries. However, sensitivity analysis (binary logistic regression models using ‘less than good SRH’ as outcomes) confirmed clear correlations compared with the results calculated by the ordinal logistic regression model (data shown in Supplemental Figure 1–1). These results indicated that the application of ordinal logistic regression models did not distort the overall analysis and supported the view that our results were robust.

### Interpretations

In Japan, socioeconomic inequalities in SRH followed the same regular gradient pattern observed in European countries and US, and we found that Japan had the smallest socioeconomic inequalities. As we discussed in the Limitations section, the high prevalence of ‘fair’ may be at least in part a product of response bias; however, the proportional odds regression analysis confirmed the small socioeconomic differences for each cut-off point (e.g. ‘less than good SRH’, or ‘bad and very bad’) among the Japanese population. Because binary logistic model analysis (Supplemental Figure 1–1) also confirmed small inequalities in Japan, we concluded that Japan had smaller inequalities in SRH than western countries.

The main results apply to a wide age range (from 30 to 79 years), but this large range may hide differences regarding socioeconomic inequalities in SRH between different age groups (e.g. between working age and retired individuals). To examine whether our findings were similar when divided by age group, we performed an additional analysis restricted to working age (30–64 years) and elderly (65–79 years) individuals. Supplemental Figure 1–2 shows the correlations of educational inequalities (low vs. high education) between the working age and elderly groups. The magnitude of educational inequalities was highly correlated between working age and the elderly (Pearson’s correlation coefficients: 0.74 (*P*<0.01) for men and 0.57 (*P*<0.01) for women), indicating that the wide age range did not affect the overall picture of socioeconomic inequalities in SRH. Although SRH worsens in tandem with aging, as we have already reported in an analysis focusing on individuals of working age (25–64 years) and the elderly (65–94 years) in Japan [32], we found that variations in inequalities in SRH between countries were more prominent than the differences in inequalities in SRH between age groups within each country.

A high prevalence of less than good SRH with small socioeconomic inequalities in Japan (shown in Figure 4(b); Japan did not follow the correlation line of European countries) may imply that small socioeconomic inequalities are partly due to relatively low SRH among high socioeconomic groups (i.e. highly educated and upper non-manual workers) for both sexes. In European countries, variations in socioeconomic inequalities in SRH mainly depend on the prevalence of low SRH among low socioeconomic status groups because the prevalence of low SRH among high socioeconomic status groups was similar across almost all European countries. We confirmed this pattern, as shown in Figure 2; the prevalence of ‘bad/very bad’ among highly educated men ranged from 1.9% (Cyprus) to 9.4% (Croatia) in Europe, whereas the prevalence was 10.4% in Japan. We found relatively low SRH even for high socioeconomic status groups among the Japanese population, a unique pattern of SRH. Further study is needed to shed more light on whether the Japanese high socioeconomic status groups have a worse objective health status than their counterparts in western countries.

Most of the studies focusing on SRH in Japan have been conducted using dichotomised outcomes, such as 0 = very good, good, or fair and 1 = bad/very bad [15, 33–37]. However, this approach may hide the full spectrum of SRH for the Japanese population because the high prevalence of ‘fair’ plays an important role. Our findings emphasise that further research should include the analysis of the prevalence of ‘fair’ as well as the prevalence of ‘bad/very bad’. This pattern (the high prevalence of ‘fair’) applies to Portugal as well because Portugal had the highest prevalence of ‘fair’ (39.2% for men and 42.6% for women) among European countries included in this study. It is worth noting that Japan and Portugal (and probably South Korea) share similar patterns of SRH, and these countries stand out as high-income countries with high life expectancy, but relatively low SRH [14]. More discussion that includes social and cultural differences between Japan and Portugal may help understand these remarkable phenomena.

The cultural context may attribute to the unique patterns observed in Japan as compared to Europe and the US. Previous studies suggested that Japan and South Korea share many features which may affect SRH [15, 16]. For example, Japanese and South Korean people more often visit doctors than individuals from other high-income countries [14]. This is partly attributed to their healthcare systems, with low barriers to medical consultation; however, low SRH may also cause more frequent doctor consultations [16]. Conversely, perceived health might be affected, and probably worsened in most cases, by repeatedly exaggerated diagnosis. The attitude of medical-seeking behaviour should be considered as an important part of understanding health issues in Japan and South Korea.

## Conclusions

Japan had relatively low SRH even for high socioeconomic status groups, and we identified smaller socioeconomic inequalities in SRH compared with those in the US and all European countries that have clear gradients in inequality. The origin of the high prevalence of lower SRH in Japan may be related to its culture, which may be similar to that in neighbouring countries (e.g. South Korea). From the perspective of comparability and data availability, global standardisation of SRH questionnaires is necessary for a more in-depth and accurate analysis. We believe that further studies of the differences and similarities between east Asian and western countries, including social-cultural aspects, are the key elements for a better understanding of global health inequalities.

## Supplemental Material

sj-docx-1-sjp-10.1177_14034948221092285 – Supplemental material for Socioeconomic inequalities in self-rated health in Japan, 32 European countries and the United States: an international comparative study Click here for additional data file. Supplemental material, sj-docx-1-sjp-10.1177_14034948221092285 for Socioeconomic inequalities in self-rated health in Japan, 32 European countries and the United States: an international comparative study by Hirokazu Tanaka, Wilma J. Nusselder, Yasuki Kobayashi and Johan P. Mackenbach in Scandinavian Journal of Public Health
