# Supplementary material for: Socioeconomic inequalities in self-rated health in Japan, 32 European countries and the United States: an international comparative study
Source: Scand J Public Health. 2022 May 10;51(8):1161–72. doi: 10.1177/14034948221092285 (PMC10642222; doi:10.1177/14034948221092285)
Supplement: sj-docx-1-sjp-10.1177_14034948221092285 – Supplemental material for Socioeconomic inequalities in self-rated health in Japan, 32 European countries and the United States: an international comparative study [file sj-docx-1-sjp-10.1177_14034948221092285.docx]

**Supplementary file**

*Tanaka et.al.* *Socioeconomic inequalities in self-rated health in Japan, 32 European countries and the United States: an international comparative study*

**Appendix Table S1-1.** Definitions of educational level

**Appendix Table S1-2.** Definitions of occupational class

**Appendix Table S1-3.** Odds ratios (OR) of lower self-rated health by educational level among men aged 30-79 years

**Appendix Table S1-4.** Odds ratios (OR) of lower self-rated health by educational level among women aged 30-79 years

**Appendix Table S1-5.** Odds ratios (OR) of lower self-rated health by occupational class among men aged 30-64 years

**Appendix Table S1-6.** Odds ratios (OR) of lower self-rated health by occupational class among women aged 30-64 years

**Appendix Figure S1-1.** Correlations of estimates between the ordinal and binary logistic regression models (sensitivity analysis)

**Appendix Figure S1-2.** Correlations of educational inequalities (low vs. high) between the working-age group and the elderly (stratified analysis by age groups)

| **Appendix Table S1-1.** Definitions of educational level | | | |
| --- | --- | --- | --- |
| Educational level | Japan | European countries* | United States |
| Low | Elementary school/Junior high school graduates | ISCED 1: Primary education | Never attended school or only kindergarten |
|  |  | ISCED 2: Lower secondary education | Grades 1 through 8 (Elementary) |
|  |  |  | Grades 9 through 11 (Some high school) |
| Middle | High school graduates | ISCED 3: Upper secondary education | Grade 12 or GED (High school graduate) |
|  | Technical professional school graduates | ISCED 4: Post-secondary non-tertiary education | College 1 year to 3 years (Some college or technical school) |
| High | 2-year college graduates | ISCED 5: Short-cycle tertiary education | College 4 years or more (College graduate) |
|  | University graduates | ISCED 6: Bachelor’s or equivalent level |  |
|  | Graduate school | ISCED 7: Master’s or equivalent level |  |
|  |  | ISCED 8: Doctoral or equivalent level |  |
| *Defined by the International Standard Classification of Education (ISCED) | | | |

| **Appendix Table S1-2.** Definitions of occupational class | | | |
| --- | --- | --- | --- |
| Occupational class | Japan* | European countries** | Correspondence to the Erikson-Goldthorpe-Portocarero scheme |
| Upper non-manual workers | (A) Administrative and managerial workers | (1) Managers | I: Higher-grade professionals, administrators and officials; managers in large industrial establishments; large proprietors |
|  | (B) Professional and engineering workers | (2) Professionals |  |
|  |  | (3) Technicians and associate professionals | II: Lower-grade professionals, administrators and officials; higher-grade technicians; managers in small business and industrial establishments; supervisors of non-manual employees |
| Lower non-manual workers | (C) Clerical workers | (4) Clerical support workers | III: Routine non-manual employees in administration and commerce; sales personnel; other rank-and-file service workers |
|  | (D) Sales workers | (5) Services and sales workers |  |
|  | (E) Service workers | - |  |
| Manual workers | (H) Manufacturing process workers | (7) Craft and related trades workers | V/VI: Lower-grade technicians; supervisors of manual workers; skilled manual workers |
|  | (I) Transport and machine operating workers | (8) Plant and machine operators and assemblers |  |
|  | (J) Construction and mining workers |  |  |
|  | (K) Carrying, cleaning, packaging, and related workers | (9) Elementary Occupations | VIIa: Semi- and unskilled manual workers (not in agriculture) |
| Farmers | (G) Agriculture forestry and fishery workers | (6) Skilled agricultural, forestry and fishery workers | IVc: Farmers and smallholders; self-employed fishermen  VIIb: Agricultural workers |
| Self-employed | Employment status: Self-employed with employees, self-employed without employees | Employment status: Self-employed with employees, self-employed without employees, family worker | IVa: Small proprietors; artisans, etc., with employees  IVb: Small proprietors, artisans, etc., without employees |
| *Defined by the Japanese Standard Occupational Classification (surveyed in the Comprehensive Survey of Living Conditions)  **Defined by the International Standard Classification of Occupations (surveyed in the EU Statistics on Income and Living Conditions) | | | |

| **Appendix Table S1-3.** Odds ratios (OR)* of lower self-rated health by educational level** among men aged 30-79 years | | | | | | | | | | | |
| --- | --- | --- | --- | --- | --- | --- | --- | --- | --- | --- | --- |
|  | | Number of included respondents | High (ISCED: 5-8) | Middle  (ISCED: 3, 4) | | | | Low  (ISCED: 1, 2) | | | |
|  |  |  |  | OR | 95% CI | | | OR | 95% CI | | |
| Japan | | 153,433 | Reference | 1.35 | 1.30 | - | 1.41 | 1.72 | 1.61 | - | 1.85 |
| Europe (pooled data)*** | | - | Reference | 1.76 | 1.69 |  | 1.84 | 2.10 | 2.01 |  | 2.20 |
|  | Finland | 4,051 | Reference | 1.94 | 1.66 | - | 2.28 | 2.40 | 1.90 | - | 3.02 |
|  | Sweden | 2,212 | Reference | 1.27 | 1.05 | - | 1.54 | 2.37 | 1.82 | - | 3.09 |
|  | Norway | 2,692 | Reference | 1.67 | 1.43 | - | 1.96 | 2.72 | 2.16 | - | 3.42 |
|  | Denmark | 2,399 | Reference | 1.55 | 1.27 | - | 1.88 | 1.96 | 1.40 | - | 2.76 |
|  | United Kingdom | 4,799 | Reference | 1.55 | 1.33 | - | 1.80 | 2.40 | 2.04 | - | 2.82 |
|  | Ireland | 3,693 | Reference | 2.00 | 1.60 | - | 2.50 | 2.72 | 2.25 | - | 3.29 |
|  | Iceland | 1,048 | Reference | 1.99 | 1.54 | - | 2.58 | 2.32 | 1.68 | - | 3.21 |
|  | Netherlands | 4,754 | Reference | 1.52 | 1.30 | - | 1.78 | 1.93 | 1.56 | - | 2.39 |
|  | Belgium | 3,886 | Reference | 1.36 | 1.17 | - | 1.58 | 2.53 | 2.11 | - | 3.03 |
|  | Luxemburg | 2,937 | Reference | 1.86 | 1.49 | - | 2.33 | 2.58 | 2.00 | - | 3.34 |
|  | Germany | 9,507 | Reference | 1.95 | 1.77 | - | 2.14 | 3.00 | 2.45 | - | 3.67 |
|  | Austria | 4,020 | Reference | 1.82 | 1.59 | - | 2.09 | 3.56 | 2.75 | - | 4.60 |
|  | Switzerland | 4,786 | Reference | 1.54 | 1.34 | - | 1.77 | 2.68 | 1.94 | - | 3.71 |
|  | France | 7,562 | Reference | 1.50 | 1.30 | - | 1.74 | 1.67 | 1.41 | - | 1.98 |
|  | Spain | 11,269 | Reference | 1.20 | 1.03 | - | 1.40 | 2.09 | 1.83 | - | 2.40 |
|  | Portugal | 8,077 | Reference | 1.74 | 1.42 | - | 2.13 | 3.48 | 2.94 | - | 4.11 |
|  | Italy | 15,151 | Reference | 1.50 | 1.32 | - | 1.71 | 2.43 | 2.14 | - | 2.76 |
|  | Greece | 13,947 | Reference | 1.41 | 1.26 | - | 1.57 | 2.10 | 1.87 | - | 2.36 |
|  | Cyprus | 3,202 | Reference | 2.09 | 1.72 | - | 2.53 | 3.86 | 3.12 | - | 4.78 |
|  | Malta | 3,546 | Reference | 1.13 | 0.88 | - | 1.44 | 2.06 | 1.66 | - | 2.55 |
|  | Slovenia | 3,208 | Reference | 2.32 | 1.94 | - | 2.77 | 4.07 | 3.19 | - | 5.19 |
|  | Croatia | 6,132 | Reference | 2.05 | 1.74 | - | 2.41 | 3.97 | 3.24 | - | 4.87 |
|  | Serbia | 5,556 | Reference | 1.92 | 1.63 |  | 2.26 | 3.03 | 2.50 |  | 3.68 |
|  | Czech Republic | 3,858 | Reference | 2.08 | 1.77 | - | 2.45 | 4.74 | 3.13 | - | 7.17 |
|  | Slovakia | 4,771 | Reference | 1.63 | 1.38 | - | 1.93 | 3.50 | 2.56 | - | 4.78 |
|  | Hungary | 5,443 | Reference | 2.32 | 1.95 | - | 2.78 | 4.18 | 3.33 | - | 5.24 |
|  | Poland | 8,538 | Reference | 2.13 | 1.86 | - | 2.44 | 3.36 | 2.78 | - | 4.06 |
|  | Bulgaria | 5,705 | Reference | 1.50 | 1.28 | - | 1.77 | 2.35 | 1.95 | - | 2.82 |
|  | Romania | 5,941 | Reference | 1.28 | 1.03 | - | 1.59 | 1.72 | 1.38 | - | 2.14 |
|  | Lithuania | 1,954 | Reference | 2.28 | 1.72 | - | 3.02 | 3.12 | 1.94 | - | 5.04 |
|  | Latvia | 3,671 | Reference | 1.89 | 1.57 | - | 2.27 | 3.10 | 2.45 | - | 3.93 |
|  | Estonia | 2,784 | Reference | 1.79 | 1.45 | - | 2.21 | 2.77 | 2.11 | - | 3.62 |
| United States**** | | 168,655 | Reference | 2.31 | 2.22 | - | 2.41 | 6.65 | 6.22 | - | 7.12 |
| *Estimates were calculated with the use of proportional odds logistic regression models controlling age category (5-years). | | | | | | | | | | | |
| **Educational level was defined by the International Standard Classification of Education (ISCED). | | | | | | | | | | | |
| *** Pooled data from 26 EU member countries except for Luxemburg and Malta as of 2016. | | | | | | | | | | | |
| ****The response scale used in the United States was asymmetrical whereas the response scale was symmetrical in Japan and European countries. | | | | | | | | | | | |
|  |  |  |  |  |  |  |  |  |  |  |  |
| 95%CI: 95% Confidence Interval | | | | | | | | | | | |

| **Appendix Table S1-4.** Odds ratios (OR)* of lower self-rated health by educational level** among women aged 30-79 years | | | | | | | | | | | |
| --- | --- | --- | --- | --- | --- | --- | --- | --- | --- | --- | --- |
|  |  | Number of included respondents | High  (ISCED: 5-8) | Middle  (ISCED: 3, 4) | | | | Low  (ISCED: 1, 2) | | | |
|  |  |  |  | OR | 95% CI | | | OR | 95% CI | | |
| Japan | | 163,491 | Reference | 1.31 | 1.26 | - | 1.37 | 1.79 | 1.65 | - | 1.95 |
| Europe (pooled data)*** | | - | Reference | 1.75 | 1.69 | - | 1.82 | 2.43 | 2.33 | - | 2.54 |
|  | Finland | 3,861 | Reference | 1.80 | 1.53 | - | 2.12 | 2.55 | 2.03 | - | 3.21 |
|  | Sweden | 2,212 | Reference | 1.53 | 1.27 | - | 1.84 | 2.40 | 1.86 | - | 3.11 |
|  | Norway | 2,517 | Reference | 1.71 | 1.44 | - | 2.03 | 2.80 | 2.22 | - | 3.54 |
|  | Denmark | 2,614 | Reference | 1.50 | 1.25 | - | 1.81 | 2.30 | 1.75 | - | 3.02 |
|  | United Kingdom | 6,017 | Reference | 1.54 | 1.35 | - | 1.76 | 2.64 | 2.32 | - | 3.01 |
|  | Ireland | 4,058 | Reference | 1.69 | 1.41 | - | 2.02 | 3.61 | 2.97 | - | 4.40 |
|  | Iceland | 1,126 | Reference | 1.57 | 1.20 | - | 2.05 | 2.62 | 1.98 | - | 3.46 |
|  | Netherlands | 5,517 | Reference | 1.63 | 1.41 | - | 1.88 | 2.54 | 2.09 | - | 3.07 |
|  | Belgium | 4,205 | Reference | 1.67 | 1.44 | - | 1.93 | 2.76 | 2.32 | - | 3.29 |
|  | Luxemburg | 3,091 | Reference | 1.83 | 1.42 | - | 2.35 | 3.56 | 2.78 | - | 4.56 |
|  | Germany | 10,749 | Reference | 1.65 | 1.47 | - | 1.84 | 2.97 | 2.55 | - | 3.46 |
|  | Austria | 4,417 | Reference | 1.62 | 1.41 | - | 1.86 | 3.90 | 3.22 | - | 4.72 |
|  | Switzerland | 5,199 | Reference | 1.20 | 1.04 | - | 1.38 | 2.51 | 2.02 | - | 3.11 |
|  | France | 8,372 | Reference | 1.55 | 1.36 | - | 1.76 | 2.42 | 2.08 | - | 2.82 |
|  | Spain | 12,127 | Reference | 1.51 | 1.31 | - | 1.74 | 2.25 | 1.98 | - | 2.55 |
|  | Portugal | 9,408 | Reference | 2.24 | 1.88 | - | 2.66 | 4.62 | 4.00 | - | 5.34 |
|  | Italy | 16,188 | Reference | 1.48 | 1.30 | - | 1.69 | 2.37 | 2.08 | - | 2.70 |
|  | Greece | 15,107 | Reference | 1.38 | 1.23 | - | 1.55 | 2.38 | 2.11 | - | 2.67 |
|  | Cyprus | 3,760 | Reference | 2.22 | 1.84 | - | 2.67 | 3.71 | 2.99 | - | 4.62 |
|  | Malta | 3,771 | Reference | 0.98 | 0.76 | - | 1.27 | 1.89 | 1.50 | - | 2.38 |
|  | Slovenia | 3,486 | Reference | 2.40 | 2.03 | - | 2.83 | 4.78 | 3.85 | - | 5.93 |
|  | Croatia | 6,731 | Reference | 1.92 | 1.63 | - | 2.26 | 3.64 | 3.04 | - | 4.35 |
|  | Serbia | 5,921 | Reference | 1.99 | 1.68 | - | 2.37 | 4.36 | 3.61 | - | 5.26 |
|  | Czech Republic | 5,809 | Reference | 2.25 | 1.93 | - | 2.61 | 4.42 | 3.51 | - | 5.55 |
|  | Slovakia | 5,730 | Reference | 1.83 | 1.57 | - | 2.14 | 3.94 | 3.14 | - | 4.93 |
|  | Hungary | 6,878 | Reference | 2.06 | 1.77 | - | 2.40 | 4.93 | 4.14 | - | 5.88 |
|  | Poland | 10,508 | Reference | 2.07 | 1.84 | - | 2.33 | 3.74 | 3.18 | - | 4.39 |
|  | Bulgaria | 6,471 | Reference | 1.69 | 1.47 | - | 1.94 | 3.21 | 2.74 | - | 3.76 |
|  | Romania | 6,481 | Reference | 1.65 | 1.34 | - | 2.04 | 2.08 | 1.69 | - | 2.57 |
|  | Lithuania | 3,473 | Reference | 2.82 | 2.29 | - | 3.48 | 5.30 | 3.69 | - | 7.61 |
|  | Latvia | 5,068 | Reference | 2.19 | 1.90 | - | 2.52 | 4.24 | 3.42 | - | 5.26 |
|  | Estonia | 4,094 | Reference | 1.93 | 1.65 | - | 2.26 | 2.60 | 2.10 | - | 3.23 |
| United States**** | | 220,634 | Reference | 2.48 | 2.38 | - | 2.58 | 8.82 | 8.29 | - | 9.38 |
| *Estimates were calculated with the use of proportional odds logistic regression models controlling age category (5-years). | | | | | | | | | | | |
| **Educational level was defined by the International Standard Classification of Education (ISCED). | | | | | | | | | | | |
| ***Pooled data from 26 EU member countries except for Luxemburg and Malta as of 2016. | | | | | | | | | | | |
| ****The response scale used in the United States was asymmetrical whereas the response scale was symmetrical in Japan and European countries. | | | | | | | | | | | |
|  |  |  |  |  |  |  |  |  |  |  |  |
| 95%CI: 95% Confidence Interval | | | | | | | | | | | |

| **Appendix Table S1-5.** Odds ratios (OR)* of lower self-rated health by occupational class** among men aged 30-64 years | | | | | | | | | | | | | | | | | | | |
| --- | --- | --- | --- | --- | --- | --- | --- | --- | --- | --- | --- | --- | --- | --- | --- | --- | --- | --- | --- |
|  | | Number of included respondents | Upper non-manual workers (I+II) | Lower non-manual workers (III) | | | | Manual workers (V+VI+VIIa) | | | | Farmers  (IVc+VIIb) | | | | Self-employed (IVa+b) | | | |
|  |  |  |  | OR | 95% CI | | | OR | 95% CI | | | OR | 95% CI | | | OR | 95% CI | | |
| Japan | | 84,357 | Reference | 1.21 | 1.16 | - | 1.27 | 1.28 | 1.23 | - | 1.34 | 1.18 | 1.07 | - | 1.29 | 1.08 | 1.02 | - | 1.15 |
| Europe (pooled data)*** | | - | Reference | 1.44 | 1.35 | - | 1.54 | 1.98 | 1.88 | - | 2.08 | 1.80 | 1.62 | - | 2.00 | 1.18 | 1.11 | - | 1.25 |
|  | Finland | 3,016 | Reference | 1.37 | 1.01 | - | 1.86 | 2.26 | 1.80 | - | 2.85 | 2.62 | 1.85 | - | 3.71 | 1.23 | 0.95 | - | 1.58 |
|  | Sweden | 1,512 | Reference | 1.66 | 1.16 | - | 2.36 | 1.64 | 1.26 | - | 2.12 | 1.49 | 0.78 | - | 2.86 | 1.48 | 1.06 | - | 2.05 |
|  | Norway | 1,946 | Reference | 1.76 | 1.29 | - | 2.41 | 2.05 | 1.65 | - | 2.57 | 2.32 | 1.38 | - | 3.90 | 1.95 | 1.35 | - | 2.84 |
|  | Denmark | 1,553 | Reference | 1.61 | 1.10 | - | 2.35 | 2.42 | 1.83 | - | 3.19 | 1.15 | 0.44 | - | 3.00 | 0.85 | 0.56 | - | 1.28 |
|  | United Kingdom | 3,249 | Reference | 1.64 | 1.31 | - | 2.05 | 1.87 | 1.55 | - | 2.26 | 1.58 | 0.91 | - | 2.73 | 0.98 | 0.73 | - | 1.31 |
|  | Ireland | 2,692 | Reference | 2.07 | 1.58 | - | 2.72 | 2.77 | 2.17 | - | 3.54 | 2.50 | 1.52 | - | 4.09 | 1.16 | 0.88 | - | 1.52 |
|  | Netherlands | 3,386 | Reference | 1.64 | 1.25 | - | 2.15 | 2.05 | 1.63 | - | 2.57 | 1.47 | 0.93 | - | 2.30 | 0.86 | 0.67 | - | 1.11 |
|  | Belgium | 2,995 | Reference | 1.34 | 1.09 | - | 1.64 | 1.97 | 1.63 | - | 2.38 | 0.69 | 0.39 | - | 1.22 | 1.16 | 0.89 | - | 1.51 |
|  | Luxemburg | 2,186 | Reference | 1.45 | 1.00 | - | 2.12 | 2.17 | 1.66 | - | 2.84 | 3.22 | 1.90 | - | 5.47 | 1.32 | 0.83 | - | 2.10 |
|  | Germany | 5,879 | Reference | 1.88 | 1.58 | - | 2.23 | 2.42 | 2.11 | - | 2.77 | 2.16 | 1.36 | - | 3.43 | 0.99 | 0.80 | - | 1.24 |
|  | Austria | 3,004 | Reference | 1.63 | 1.29 | - | 2.05 | 2.57 | 2.14 | - | 3.09 | 2.84 | 1.96 | - | 4.10 | 1.12 | 0.88 | - | 1.43 |
|  | Switzerland | 3,628 | Reference | 1.72 | 1.30 | - | 2.26 | 2.42 | 1.92 | - | 3.06 | 1.26 | 0.94 | - | 1.69 | 1.25 | 0.92 | - | 1.70 |
|  | France | 5,642 | Reference | 1.12 | 0.87 | - | 1.43 | 1.71 | 1.46 | - | 2.01 | 1.84 | 1.42 | - | 2.38 | 1.15 | 0.89 | - | 1.48 |
|  | Spain | 8,609 | Reference | 1.43 | 1.18 | - | 1.72 | 2.02 | 1.74 | - | 2.35 | 2.53 | 1.32 | - | 4.84 | 1.61 | 1.34 | - | 1.95 |
|  | Portugal | 5,910 | Reference | 1.87 | 1.53 | - | 2.29 | 2.77 | 2.35 | - | 3.28 | 3.59 | 2.64 | - | 4.88 | 2.03 | 1.64 | - | 2.52 |
|  | Italy | 11,088 | Reference | 1.22 | 1.03 | - | 1.46 | 1.59 | 1.38 | - | 1.83 | 2.04 | 1.50 | - | 2.75 | 1.22 | 1.05 | - | 1.41 |
|  | Greece | 9,867 | Reference | 1.47 | 1.23 | - | 1.75 | 1.71 | 1.47 | - | 1.99 | 1.55 | 1.26 | - | 1.91 | 1.57 | 1.34 | - | 1.83 |
|  | Cyprus | 2,305 | Reference | 2.37 | 1.80 | - | 3.13 | 2.92 | 2.32 | - | 3.68 | 2.72 | 1.57 | - | 4.72 | 2.82 | 2.11 | - | 3.77 |
|  | Malta | 2,472 | Reference | 1.32 | 1.01 | - | 1.73 | 1.78 | 1.42 | - | 2.24 | 2.04 | 1.03 | - | 4.03 | 1.20 | 0.91 | - | 1.57 |
|  | Slovenia | 2,313 | Reference | 1.56 | 1.17 | - | 2.08 | 2.17 | 1.76 | - | 2.68 | 1.59 | 1.04 | - | 2.44 | 1.40 | 1.03 | - | 1.89 |
|  | Croatia | 4,394 | Reference | 1.34 | 1.09 | - | 1.66 | 1.66 | 1.39 | - | 1.98 | 1.93 | 1.36 | - | 2.75 | 1.27 | 0.97 | - | 1.67 |
|  | Serbia | 3,907 | Reference | 1.42 | 1.09 | - | 1.85 | 1.84 | 1.49 | - | 2.29 | 1.90 | 1.42 | - | 2.55 | 1.08 | 0.81 | - | 1.44 |
|  | Czech Republic | 2,554 | Reference | 1.75 | 1.27 | - | 2.41 | 2.09 | 1.71 | - | 2.56 | 1.88 | 1.06 | - | 3.32 | 1.01 | 0.80 | - | 1.28 |
|  | Slovakia | 3,726 | Reference | 1.81 | 1.41 | - | 2.34 | 2.19 | 1.81 | - | 2.65 | 2.09 | 1.28 | - | 3.41 | 1.36 | 1.06 | - | 1.75 |
|  | Hungary | 4,036 | Reference | 2.08 | 1.58 | - | 2.73 | 2.31 | 1.88 | - | 2.83 | 2.04 | 1.43 | - | 2.90 | 1.20 | 0.86 | - | 1.67 |
|  | Poland | 6,443 | Reference | 1.84 | 1.45 | - | 2.32 | 2.28 | 1.94 | - | 2.67 | 2.21 | 1.80 | - | 2.72 | 1.25 | 0.99 | - | 1.57 |
|  | Bulgaria | 4,023 | Reference | 1.58 | 1.20 | - | 2.07 | 1.86 | 1.51 | - | 2.29 | 1.31 | 0.91 | - | 1.87 | 1.24 | 0.91 | - | 1.68 |
|  | Romania | 4,184 | Reference | 1.05 | 0.76 | - | 1.45 | 1.56 | 1.25 | - | 1.94 | 0.93 | 0.71 | - | 1.23 | 1.50 | 1.09 | - | 2.07 |
|  | Lithuania | 1,435 | Reference | 1.51 | 0.81 | - | 2.80 | 2.26 | 1.59 | - | 3.24 | 3.13 | 1.87 | - | 5.26 | 2.02 | 1.23 | - | 3.32 |
|  | Latvia | 2,775 | Reference | 1.56 | 1.11 | - | 2.21 | 2.05 | 1.67 | - | 2.52 | 1.83 | 1.32 | - | 2.54 | 1.12 | 0.82 | - | 1.53 |
|  | Estonia | 2,054 | Reference | 2.15 | 1.33 | - | 3.50 | 3.02 | 2.32 | - | 3.93 | 3.27 | 1.82 | - | 5.87 | 1.28 | 0.88 | - | 1.88 |
| *Estimates were calculated with the use of proportional odds logistic regression models controlling age category (5-years). | | | | | | | | | | | | | | | | | | | |
| **Occupational class was defined by the Erikson-Goldthorpe-Portocarero scheme. | | | | | | | | | | | | | | | | | | | |
| ***Pooled data from 26 EU member countries except for Luxemburg and Malta as of 2016. | | | | | | | | | | | | | | | | | | | |
| 95%CI: 95% Confidence Interval | | | | | | | | | | | | | | | | | | | |

| **Appendix Table S1-6.** Odds ratios (OR)* of lower self-rated health by occupational class** among women aged 30-64 years | | | | | | | | | | | | | | | | | | | |
| --- | --- | --- | --- | --- | --- | --- | --- | --- | --- | --- | --- | --- | --- | --- | --- | --- | --- | --- | --- |
|  | | Number of included respondents | Upper non-manual workers (I+II) | Lower non-manual workers (III) | | | | Manual workers (V+VI+VIIa) | | | | Farmers  (IVc+VIIb) | | | | Self-employed (IVa+b) | | | |
|  |  |  |  | OR | 95% CI | | | OR | 95% CI | | | OR | 95% CI | | | OR | 95% CI | | |
| Japan | | 76,515 | Reference | 1.13 | 1.08 | - | 1.18 | 1.36 | 1.28 | - | 1.44 | 1.29 | 1.14 | - | 1.44 | 0.87 | 0.78 | - | 0.97 |
| Europe (pooled data)*** | | - | Reference | 1.41 | 1.35 | - | 1.48 | 2.26 | 2.14 | - | 2.38 | 1.87 | 1.72 | - | 2.05 | 1.08 | 1.01 | - | 1.16 |
|  | Finland | 2,817 | Reference | 1.81 | 1.48 | - | 2.22 | 2.94 | 2.11 | - | 4.08 | 3.46 | 2.18 | - | 5.49 | 1.41 | 1.05 | - | 1.90 |
|  | Sweden | 1,486 | Reference | 1.86 | 1.48 | - | 2.34 | 1.97 | 1.31 | - | 2.98 | 1.14 | 0.29 | - | 4.47 | 0.61 | 0.41 | - | 0.93 |
|  | Norway | 1,825 | Reference | 1.92 | 1.55 | - | 2.36 | 3.19 | 2.14 | - | 4.77 | 1.61 | 0.89 | - | 2.89 | 1.33 | 0.77 | - | 2.31 |
|  | Denmark | 1,710 | Reference | 1.68 | 1.33 | - | 2.12 | 2.19 | 1.49 | - | 3.22 | 2.15 | 0.28 | - | 16.4 | 0.53 | 0.28 | - | 0.99 |
|  | United Kingdom | 4,265 | Reference | 1.63 | 1.42 | - | 1.89 | 2.66 | 2.16 | - | 3.27 | 0.62 | 0.20 | - | 1.89 | 1.18 | 0.96 | - | 1.46 |
|  | Ireland | 2,862 | Reference | 1.33 | 1.03 | - | 1.70 | 2.47 | 1.96 | - | 3.11 | 1.77 | 1.32 | - | 2.37 | 1.06 | 0.72 | - | 1.58 |
|  | Netherlands | 3,909 | Reference | 1.72 | 1.44 | - | 2.05 | 2.51 | 1.80 | - | 3.49 | 1.62 | 0.73 | - | 3.59 | 0.95 | 0.75 | - | 1.20 |
|  | Belgium | 3,008 | Reference | 1.56 | 1.31 | - | 1.85 | 2.48 | 2.02 | - | 3.04 | 1.88 | 0.93 | - | 3.81 | 1.02 | 0.74 | - | 1.39 |
|  | Luxemburg | 2,078 | Reference | 2.05 | 1.55 | - | 2.70 | 3.38 | 2.47 | - | 4.64 | 3.04 | 0.91 | - | 10.2 | 1.11 | 0.67 | - | 1.82 |
|  | Germany | 6,803 | Reference | 1.44 | 1.28 | - | 1.62 | 2.38 | 2.01 | - | 2.82 | 1.35 | 0.65 | - | 2.79 | 0.77 | 0.56 | - | 1.04 |
|  | Austria | 3,210 | Reference | 1.55 | 1.32 | - | 1.83 | 3.41 | 2.75 | - | 4.24 | 1.79 | 1.25 | - | 2.58 | 1.16 | 0.85 | - | 1.57 |
|  | Switzerland | 3,854 | Reference | 1.52 | 1.29 | - | 1.79 | 2.09 | 1.54 | - | 2.83 | 1.02 | 0.59 | - | 1.78 | 0.91 | 0.69 | - | 1.20 |
|  | France | 5,964 | Reference | 1.41 | 1.21 | - | 1.64 | 2.04 | 1.72 | - | 2.40 | 1.86 | 1.38 | - | 2.52 | 0.91 | 0.71 | - | 1.16 |
|  | Spain | 8,623 | Reference | 1.46 | 1.26 | - | 1.70 | 2.25 | 1.91 | - | 2.63 | 1.92 | 1.36 | - | 2.70 | 1.35 | 1.04 | - | 1.76 |
|  | Portugal | 6,502 | Reference | 2.04 | 1.74 | - | 2.40 | 3.54 | 3.01 | - | 4.18 | 5.19 | 3.50 | - | 7.70 | 1.65 | 1.29 | - | 2.11 |
|  | Italy | 10,505 | Reference | 1.15 | 1.01 | - | 1.31 | 1.74 | 1.46 | - | 2.07 | 2.29 | 1.63 | - | 3.22 | 1.24 | 1.06 | - | 1.46 |
|  | Greece | 8,745 | Reference | 1.38 | 1.20 | - | 1.58 | 2.06 | 1.73 | - | 2.44 | 1.82 | 1.48 | - | 2.23 | 1.37 | 1.14 | - | 1.63 |
|  | Cyprus | 2,589 | Reference | 1.98 | 1.59 | - | 2.47 | 2.44 | 1.92 | - | 3.09 | 3.55 | 0.07 | - | 172 | 1.67 | 1.19 | - | 2.34 |
|  | Malta | 2,241 | Reference | 1.26 | 0.99 | - | 1.60 | 2.12 | 1.63 | - | 2.77 | 2.16 | 0.47 | - | 9.84 | 1.15 | 0.80 | - | 1.67 |
|  | Slovenia | 2,421 | Reference | 1.86 | 1.51 | - | 2.28 | 3.18 | 2.56 | - | 3.96 | 2.86 | 1.47 | - | 5.58 | 1.61 | 1.05 | - | 2.47 |
|  | Croatia | 4,237 | Reference | 1.62 | 1.35 | - | 1.95 | 2.66 | 2.20 | - | 3.21 | 3.65 | 2.31 | - | 5.74 | 0.86 | 0.58 | - | 1.29 |
|  | Serbia | 3,460 | Reference | 1.70 | 1.37 | - | 2.11 | 2.87 | 2.30 | - | 3.58 | 3.84 | 2.70 | - | 5.46 | 1.32 | 0.93 | - | 1.87 |
|  | Czech Republic | 3,767 | Reference | 1.71 | 1.45 | - | 2.01 | 2.89 | 2.39 | - | 3.48 | 2.34 | 1.34 | - | 4.10 | 1.13 | 0.88 | - | 1.45 |
|  | Slovakia | 4,117 | Reference | 1.40 | 1.16 | - | 1.68 | 2.12 | 1.74 | - | 2.60 | 1.80 | 1.03 | - | 3.14 | 0.97 | 0.73 | - | 1.28 |
|  | Hungary | 4,644 | Reference | 1.70 | 1.40 | - | 2.06 | 3.43 | 2.88 | - | 4.09 | 2.31 | 1.66 | - | 3.20 | 0.89 | 0.62 | - | 1.26 |
|  | Poland | 7,486 | Reference | 1.48 | 1.28 | - | 1.72 | 2.45 | 2.10 | - | 2.86 | 2.11 | 1.77 | - | 2.53 | 1.05 | 0.81 | - | 1.36 |
|  | Bulgaria | 4,020 | Reference | 1.66 | 1.37 | - | 2.01 | 2.90 | 2.38 | - | 3.53 | 2.04 | 1.41 | - | 2.94 | 1.33 | 0.88 | - | 1.99 |
|  | Romania | 3,750 | Reference | 1.53 | 1.19 | - | 1.96 | 2.49 | 1.98 | - | 3.13 | 1.28 | 0.98 | - | 1.67 | 1.34 | 0.87 | - | 2.08 |
|  | Lithuania | 2,395 | Reference | 2.26 | 1.69 | - | 3.01 | 3.62 | 2.70 | - | 4.87 | 3.77 | 2.23 | - | 6.37 | 1.79 | 1.08 | - | 2.98 |
|  | Latvia | 3,315 | Reference | 1.92 | 1.60 | - | 2.30 | 2.87 | 2.35 | - | 3.49 | 2.36 | 1.45 | - | 3.83 | 1.08 | 0.78 | - | 1.51 |
|  | Estonia | 2,889 | Reference | 1.66 | 1.33 | - | 2.05 | 3.26 | 2.61 | - | 4.08 | 2.99 | 1.66 | - | 5.37 | 1.81 | 1.15 | - | 2.84 |
| *Estimates were calculated with the use of proportional odds logistic regression models controlling age category (5-years). | | | | | | | | | | | | | | | | | | | |
| **Occupational class was defined by the Erikson-Goldthorpe-Portocarero scheme. | | | | | | | | | | | | | | | | | | | |
| ***Pooled data from 26 EU member countries except for Luxemburg and Malta as of 2016. | | | | | | | | | | | | | | | | | | | |
| 95%CI: 95% Confidence Interval | | | | | | | | | | | | | | | | | | | |

**Appendix Figure S1-1.** Correlations of estimates between the ordinal and binary logistic regression models (sensitivity analysis)

**Appendix Figure S1-2.** Correlations of educational inequalities (low vs. high) between the working-age group and the elderly (stratified analysis by age groups); Pearson’s correlation coefficients: 0.74 (*p* < 0.01) for men and 0.57 (*p* < 0.01) for women.
